# A Comparative Study of the Repair Bond Strength of New Self-Adhesive Restorative Materials With a Resin Composite Material

**DOI:** 10.7759/cureus.44309

**Published:** 2023-08-29

**Authors:** Sanem Ozaslan, Ozge Celiksoz, Hatice Tepe, Begum Tavas, Batu Can Yaman

**Affiliations:** 1 Department of Restorative Dentistry, Eskisehir Osmangazi University Faculty of Dentistry, Eskisehir, TUR

**Keywords:** resin composite, repair, microshear bond strength, glass hybrid restorative, alkasite

## Abstract

Aim: The aim of the present study is to compare the repair bond strengths (RBSs) of Cention-N (light-cure and self-cure modes), Equia Forte HT Fil and a nanohybrid resin composite.

Materials and methods: Equia Forte HT Fil (GC, Tokyo, Japan), Cention-N (Ivoclar Vivadent, Schaan, Liechtenstein) and Filtek Z550 (3M ESPE, St Paul, MN, USA) were used in this study. Equia Forte HT Fil (EQF), Cention-N self-cure (CSC), Cention-N light-cure (CLC) and Filtek Z550 (Z550) groups were formed. A total of 40 samples were prepared; 10 samples in each group (n = 10). After the polymerization was completed according to the manufacturer's instructions, the samples were polished with OptiDisc (Kerr Corporation, Orange, USA) for 5 s each, from extra-course to extra-fine. After all samples were stored in 37°C water for 24 h, 10,000 cycles of brushing simulator and thermal cycles were applied to the samples. The samples were prepared in accordance with the selected repair protocol, and microshear bond strength (µSBS) test was performed. Fracture analysis on debonded surfaces was visualized by scanning electron microscopy. The conformity of the data to normal distribution was analyzed by the Shapiro-Wilk test. Multiple comparisons were performed using Dunn's test.

Results: Z550 showed significantly higher µSBS as compared to the other three groups. There is no difference between CSC, CLC and EQF.

Conclusion: The use of Cention-N's self-cure or light-cure mode did not affect the RBS values. The RBS values of Cention-N and Equia Forte HT Fil materials are lower than those of the composite resin material.

## Introduction

To keep teeth functional for a lifetime, minimally invasive dentistry was developed and is now widely accepted [1]. To safeguard the teeth from the repetitive restoration cycle, one of the commonly held beliefs regarding this concept is that defective restorations should be repaired as opposed to being replaced. If restorations cannot be repaired due to serious and widespread problems such as extensive caries or the presence of cracked cusps adjacent to the existing restoration, they must be replaced. Typically, these replacements result in the loss of more tooth structure [2]. There are numerous advantages to repairing a restoration as opposed to replacing it, including preservation of tooth structure and pulp, generally no need for local anesthesia, reduced treatment cost and time, improved patient tolerance, and slowing the "restorative death spiral" [3].

The disadvantages of resin composites, such as requiring technical precision in the application stages, difficulty in placing more than 2 mm, shrinkage during polymerization and unknown polymerization depth, push manufacturers to produce new materials [4]. In line with the solution to these problems, many companies producing dental materials have worked on 'self-adhesive' systems to simplify the bonding procedures of the restorative material to the dental tissue. Self-adhesive materials bond chemically to hydroxyapatite by monomers that can etch enamel and dentin [5]. In addition, some self-adhesive materials developed have the feature of 'releasing fluoride ions' against caries, which is still a common problem today. 

Due to the advances in the chemical and mechanical performance of glass ionomer cement (GIC), their use in restorative dentistry has become more widespread. The most recently developed materials in GIC technology are glass hybrid restorative (GHR) materials. Equia Forte Fil (GC, Japan) was first developed in 2015. In 2019, the latest version of these materials, Equia Forte HT Fil (GC, Japan), was produced. According to the manufacturer, the matrix of this new system, which it refers to as GHR, consists of fluoroaluminosilicate particles and fillers of various sizes comparable to hybrid composite resins. This material is a self-adhesive, chemically cured product that can be used permanently in posterior restorations [6,7]. It has enhanced flexural fatigue resistance, fracture toughness and flexural strength [8]. Thanks to these advanced properties, GHR is finding increasing applications.

And just recently, Cention-N (Ivoclar Vivadent, Liechtenstein) was introduced as a new alkasite material. Alkasite is essentially a subgroup of resin composite. It is an aesthetically appealing, tooth-colored material with exceptional flexural strength. The material can release fluoride ions and neutralize acids thanks to alkaline fillers. It can be used as a light-cure, self-cure, and volume placement material, with or without an adhesive system [6,8].

As the use of self-adhesive materials that release fluoride ions increases, so will the need for repair. Although there are some studies [9,10] on the repair of GHR, according to the authors' knowledge, there are no studies in the literature on the repair using the latest version, Equia Forte HT Fil, with resin composite. Furthermore, to the best of the authors' knowledge, there are no studies in the literature on the repair efficacy of Cention-N material with resin composite.

The aim of the present study is to compare the repair bond strength (RBS) of Cention-N (light-cure and self-cure modes), Equia Forte HT Fil and a nanohybrid resin composite. The null hypothesis tested in the present study is that there will be no difference in RBS between the groups tested.

## Materials and methods

**Figure 1:**
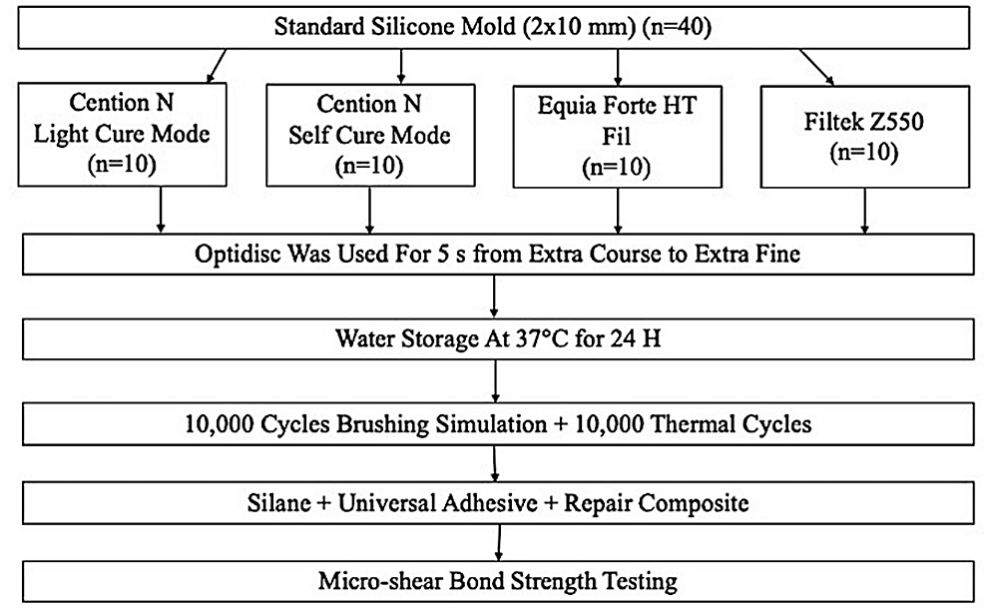
Flow diagram of the experimental design

Equia Forte HT Fil, Cention-N and Filtek Z550 (3M ESPE, USA) were used in the present study. Equia Forte HT Fil (EQF), Cention-N self-cure (CSC), Cention-N light-cure (CLC) and Filtek Z550 (Z550) groups were formed. The composition, manufacturers and Lot Number of the study materials are detailed in Table 1. The flow diagram is shown in Figure 1.

**Table 1:** The compositions and manufacturers of the materials used Bis-EMA: Bisphenol A ethoxylate dimethacrylate; Bis-GMA: Bisphenol A glycerolate dimethacrylate; PEGDMA: Polyethylene glycol dimethacrylate; TEGDMA: Triethylene glycol dimethacrylate; UDMA: Urethane-dimethacrylate.

Material	Manufacturer (Lot no)	Composition
Cention-N	Ivoclar-Vivadent, Liechtenstein (Z0054T)	Liquid: UDMA, tricyclodecan dimethanol dimethacrylate, tetramethyl-xylene diurethane dimethacrylate, polyethylene glycol 400 dimethacrylate, ivocerin, hydroxyperoxide. Powder: Barium aluminum silicate glass, ytterbium trifluoride, isofiller, calcium barium aluminum fluorosilicate glass, calcium fluorosilicate glass
EQUIA Forte HT Fil	GC, Japan (2012181)	Liquid: Polybasic carboxylic acid, water. Powder: Fluoroaluminosilicate glass, polyacrylic acid, iron oxide
EQUIA Forte Coat	GC, Japan (1804021)	Methyl methacrylate, colloidal silica, camphorquinone, urethane methacrylate, phosphoric ester monomer
Filtek Z550	3M ESPE, USA (N728631)	Bis-GMA, UDMA, Bis-EMA, TEGDMA, PEGDMA, Surface-modified zirconia/silica fillers, non-agglomerated/non-aggregated surface-modified silica particles 2 nm
Neo Spectra ST HV	Dentsply Sirona, Germany (2211000700)	Methacrylate-modified polysiloxane (organically modified ceramic) dimethacrylate resins, ethyl 1-4 (dimethyl amino) benzoate and bis (4-methyl-phenyl) iodonium hexafluorophosphate 78%-80% by weight spherical prepolymerized SphereTEC fillers, non-agglomerated barium glass and ytterbium fluoride
Prime&Bond Universal	Dentsply Sirona, Germany (2112000581)	Phosphoric acid-modified acrylate resin, multifunctional acrylate, bifunctional acrylate, acid acrylate, isopropanol, water, initiator
Clearfil Ceramic Primer Plus	Kuraray Noritake Dental Inc., Japan (CJ0032)	Ethanol (%80), 3-trimethoxysilylpropyl methacrylate (%5), 10-methacryloyloxydecyl dihydrogen phosphate

Preparing the samples

The number of samples to be included in this study was determined in the G*Power software (version 3.1.9.4). A total of 10 samples were planned per group (power: 0.80, α:0.05, effect size: 2.4). Using standard 2 mm depth and 10 mm diameter silicone molds, a total of 40 samples, 10 from each group, were fabricated. For sample surface standardization, OptiDisc (Kerr Corporation, USA) was used for each sample from extra-course to extra-fine for 5 s per disc. After forming a uniform surface on all samples, they were stored in 37°C distilled water for 24 h. The protocols for sample preparation and curing are detailed in Table 2.

**Table 2:** The protocols for sample preparation and curing LED: Light-emitting diode, CSC: Cention-N self-cure, CLC: Cention-N light-cure, EQF: Equia Forte HT Fil.

Material	Curing Protocol Used
CSC	According to the manufacturer's instructions, each sample of Cention-N was manually mixed at a powder-to-liquid ratio of 1:1. The upper surface was subsequently covered with Mylar tape and a glass slab using finger pressure. The samples were removed from the mold after 4 min at room temperature.
CLC	According to the manufacturer's instructions, each sample of Cention-N was manually mixed at a powder-to-liquid ratio of 1:1. The surface was then covered with Mylar tape and a glass slab by applying finger pressure. The samples' upper surfaces were polymerized for 20 s with an LED light device (VALO Cordless, Ultradent Products Inc., USA) at 954 mW/cm^2^ irradiance.
EQF	For each sample, one EQF capsule was mechanically mixed for 10 s as per the manufacturer's instructions. After 2.5 min at room temperature, the coating was applied. It was polymerized for 20 s with the same LED device.
Z550	For the preparation of resin composite samples, 3M Filtek Z550 resin composite material was placed in silicone molds with a hand instrument. Then, the upper surface was covered with Mylar tape and a glass slab under finger pressure. Polymerized for 20 s with the same LED device.

Aging procedure

All samples were subjected to 10,000 cycles of simulated brushing with toothpaste (Sensodyne Repair & Protect, GlaxoSmithKline, UK) diluted 1/3 by volume under a load of 250 g using an Esetron MTB 100 (MOD Dental, Esetron Smart Robotechnologies, Ankara, Turkey) device with an 18 mm movement diameter. After the brushing simulation, each sample was subjected to 10,000 cycles in an Esetron (MOD Dental, Esetron Smart Robotechnologies, Ankara, Turkey) thermal cycler with a water tank between 5°C and 55°C, with a 30 s dwell time and 10 s transfer time at each temperature.

Experimental repair protocol

Using a high-speed handpiece and bur with a particle size of 46 nm, the samples were roughened with a five-back-and-forth motion for 10 s with air-water cooling [11]. Different burs were used for each sample. Afterward, the samples were then rinsed and dried.

Silane (Clearfil Ceramic Primer Plus) was applied to the samples with an applicator for 20 s and dried with an air spray for 10 s, according to the manufacturer's instructions. Universal adhesive (Prime&Bond Universal) was agitated for 20 s and then gently air-dried for 5 s. It was polymerized for 20 s with a light-emitting diode (LED) light device (VALO Cordless, Ultradent Products Inc., USA) at 954 mW/cm^2^ irradiance. The samples were standardized using Tygon tubes (1.6x2 mm). After the resin, composite material (Neo Spectra ST HV) was filled into the Tygon tube with a hand instrument, and it was polymerized with light for 20 s. After removing the Tygon tubes, an additional 40 s of circumferential polymerization was applied. All the samples were stored in water at 37°C for 24 h.

Microshear bond strength test and fracture analysis

Microshear bond strength (µSBS) test was conducted using a universal testing machine (MOD Dental MIC-101, Esetron Smart Robotechnologies, Ankara, Turkey) and a metal frame with a crosshead speed of 0.5 mm/min and a 50 kg load cell until the bond failed. Values were recorded in Newton (N) and then converted to megapascals (MPa) (MPa=N/Surface area). Using a stereomicroscope (Model M80; Leica Microsystems Ltd., Switzerland), fractures on the debonded samples were analyzed at x30 magnification and classified as adhesive, cohesive or mixed (combination adhesive and cohesive) failures.

Scanning electron microscopy analysis

The scanning electron microscopy (SEM) analysis of the samples was performed with Hitachi Regulus 8230 (Hitachi High-Technologies Corporation, Japan) at 10.0 kV. The samples were examined at x50 magnification. 

Statistical analysis

Data was analyzed with Statistical Package for the Social Sciences (SPSS) (v23, IBM SPSS Statistics, USA). Compliance with normal distribution was analyzed by the Shapiro-Wilk test. Non-normally distributed parameters were compared using the Kruskal-Wallis H test, and multiple comparisons were made using Dunn's test. The results of the analyses were presented as mean ± standard deviation and median (minimum-maximum). The significance level was taken as p<0.05.

## Results

The group with the highest µSBS value was Z550 (27.39 MPa ± 3.66), while the group with the lowest µSBS value was CSC (10.61 MPa ± 2.76). The values measured in the Z550 group were statistically different from the CSC, CLC and EQF groups. There is no difference between CSC, CLC and EQF (p>0.05) (Table 3).

**Table 3:** µSBS values (MPa) of the groups *Kruskal Wallis H test; Mean ± Standard Deviation; Median (Q1-Q3); Q1: percentile 25 Q3: percentile 75; a-b: Values with the same superscript letter are not significantly different. µSBS: microshear bond strength, CSC: Cention-N self-cure, CLC: Cention-N light-cure, EQF: Equia Forte HT Fil.

Groups	Mean ± SD	Median (Q1-Q3)	Test Statistic	p*
CSC	10.61 ± 2.76	8.86 (8.56-14.33)^b^	22.986	<0.001
CLC	12.18 ± 3	11.35 (9.55-13.93)^b^		
EQF	11.92 ± 3.19	13.14 (8.36-13.93)^b^		
Z550	27.39 ± 3.66	25.98 (25.28-29.86)^a^		

**Figure 2:**
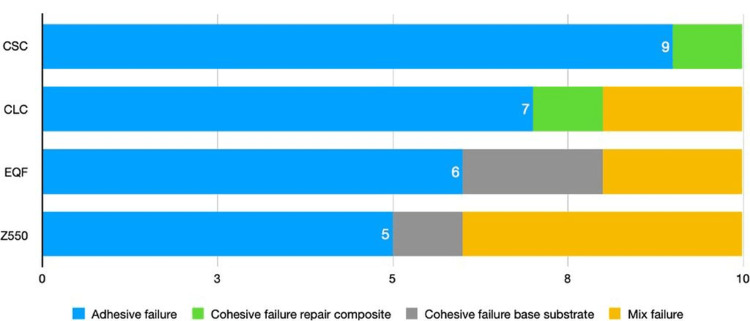
Failure types by groups CSC: Cention-N self-cure, CLC: Cention-N light-cure, EQF: Equia Forte HT Fil.

**Figure 3:**
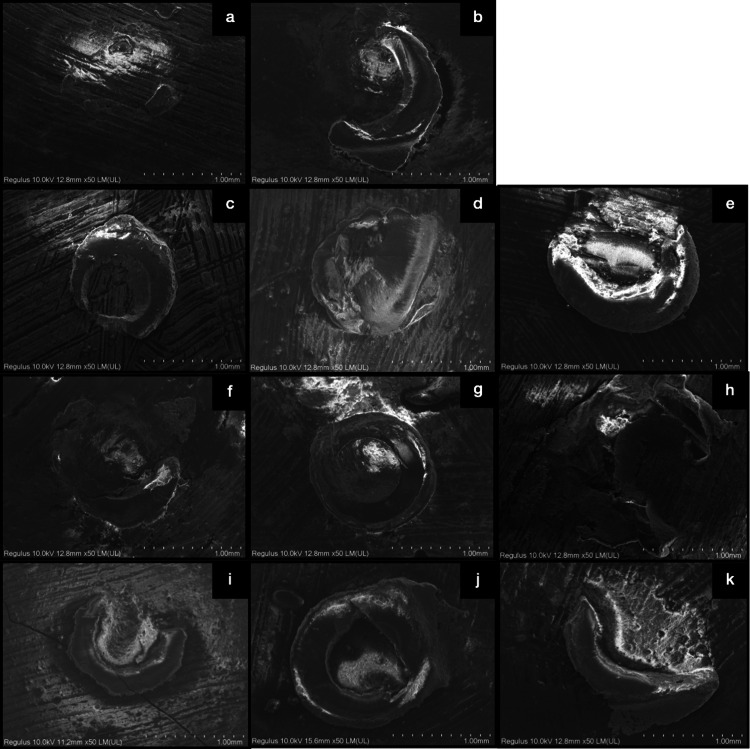
SEM images of groups according to failure types CSC group adhesive failure (a), cohesive failure repair composite (b); z550 group adhesive failure (c), cohesive failure base substrate (d), mix failure (e); CLC group adhesive failure (f), cohesive failure repair composite (g), mix failure (h); EQF group adhesive failure (i), cohesive failure base substrate (j) and mix failure (k). SEM: Scanning electron microscopy, CSC: Cention-N self-cure, CLC: Cention-N light-cure, EQF: Equia Forte HT Fil.

The failure type observed most frequently across all categories was adhesive failure. Failure modes are listed in Figure 2, and SEM images of failure types are demonstrated in Figure 3.

## Discussion

The present study compared the RBS of the current self-adhesive restorative materials Cention-N (self-cure and light-cure modes) and Equia Forte HT Fil with the RBS of a nanohybrid resin composite. While the Z550 group showed the highest RBS value, no significant difference was found between the CSC, CLC and EQF groups. Within this result, the null hypothesis was rejected.

The more intraoral factors can be reflected in the in-vitro experimental setting, the stronger the clinical predictions are expected to be. Different aging procedures are applied to dental materials to simulate intraoral conditions. Thermal aging is a frequently preferred method of aging [12]. Toothbrushing cycles also cause aging by causing changes in the surface of the material [13]. Brushing simulation and thermal aging were applied together to simulate intraoral conditions on the materials tested in the present study. Numerous researchers have adopted the theory that 10,000 cycles correspond to one year of brushing and thermal intraoral function [14-18].

Choosing to repair restorations as opposed to replacing them is minimally invasive, reduces the risk of pulp injury and reduces treatment costs [19]. The bond strength depends on the substrate's restorative material more than the selected repair material. However, dental practitioners performing the repair rarely know the substrate of the restoration. Consequently, it is challenging to forecast the clinical success of the repair [20]. Novel self-adhesive restorative materials may be clinically indistinguishable from resin restorations in the mouth due to their physical appearance characteristics similar to resin composites. In the present study, the substrate material was assumed to be an unknown resin composite. Therefore, resin composite repair procedures were applied, and a random nanohybrid resin composite was used as the repair material.

Before performing the repair procedures, a mechanical roughening of the surface of the old restoration is indicated as one of the most important steps [21]. Surface treatment methods used include air abrasion, diamond bur, hydrofluoric or phosphoric acid and laser. Despite these advancements in restoration protocols, there is still no consensus regarding the optimal surface preparation method. Among these techniques, diamond burs surface roughening is a simple, cost-effective, clinically applicable method that requires no additional equipment or chemicals [22]. Therefore, diamond burs were utilized for surface treatment in the present study.

It has been reported that the application of silane before adhesive application increases the RBS value of resin composites. In addition, they improve surface wettability by altering surface energy [23]. The 10-methacryloyloxydecyl dihydrogen phosphate (MDP) monomer allows universal adhesives to bond to a wide range of substrates, including metals, glass ceramics and zirconia making them popular in clinical practice [24]. In the present study, in addition to surface treatment with a diamond bur, repair procedures were carried out by applying silane and a universal adhesive to all samples.

Shear bond strength (SBS), µSBS and microtensile bond strength (µTBS) tests are used to evaluate RBS. Premature micro-cracking of samples may occur during sectioning for µTBS testing [25]. The test protocol and sample preparation for the µSBS test are simpler than those for the µTBS test. It has been reported that a smaller sample diameter in µSBS tests reduces the probability of stress formation and the error rate. Therefore, the µSBS test is more recommended than the SBS [26].

Some studies recommend a bond strength for resin composite repairs of between 15 MPa and 25 MPa. These values are clinically acceptable thresholds for the bond strength between the resin composite and dentin [27]. The median MPa values obtained in the CSC, CLC and EQF groups in the present study fell below the level considered to be clinically acceptable. In line with expectations, 25.98 MPa was detected in the Z550 group over the threshold value.

While there are no studies in the literature on the repair efficacy of Equia Forte HT Fil material, there are studies on Equia Forte Fil, the previous version of the GHR materials. These studies may provide some insight when evaluating the results of the present study. In a study to evaluate the RBS of resin composite and GHR material (Equia Forte Fil) with resin composite using the SBS test method, various surface treatment methods were tested [28]. For all surface treatment methods, the RBS of the resin composite was higher than that of the GHR. Although the methods of the aforementioned study and the present study are different, the results obtained are mutually supportive.

Although Cention-N is classified as an alkacid, it is a new, tooth-colored aesthetic material belonging to a subgroup of composite resins [29]. As a result, the RBS of aged Cention-N was not expected to be lower than that of the resin composite. In addition, although studies comparing the bond strength of different polymerization modes of Cention-N to dentin are available in the literature [30,31], there are no studies comparing the RBS.

It was reported that more cohesive and mixed failures were observed in macro-bond strength tests [32]. However, due to the reduction of surface area in µSBS tests, force can be applied from a more specific area targeting the entire interface, and adhesive failures are expected to be more common [32,33]. Although many studies in the literature evaluate all failure types, some researchers have suggested that only adhesive failures or mixed failures with minor (<10%) resin or dentin involvement should be considered in bond strength calculations for more reliable results [32]. In the present study, although all failure types were considered, adhesive failures were more common than cohesive failures, as expected. In addition, similar to the literature, groups with low µSBS values generally showed adhesive failures, while groups with high µSBS values showed more cohesive and mixed failures [11-12].

The limitations of this study are the evaluation of bond strength 24 h after repair and the fact that the study was performed under in vitro conditions. Long-term clinical studies are needed to evaluate the long-term bond strength and clinical performance of these materials.

## Conclusions

Within the limitations of the present study, there was no difference in µSBS between the self-cured and light-cured groups of Cention-N when repaired with resin composite. The resin composite group showed the highest µSBS value. The Cention-N and Equia Forte HT Fil groups had similar µSBS values. Further observational and randomized clinical studies are required to determine the repair capacity of self-adhesive restorative materials.
